# Supplementary material for: Mechanistic understanding of in vivo protein corona formation on polymeric nanoparticles and impact on pharmacokinetics
Source: Nat Commun. 2017 Oct 3;8:777. doi: 10.1038/s41467-017-00600-w (PMC5626760; doi:10.1038/s41467-017-00600-w)
Supplement: Supplementary file 1 — Supplementary Information [file 41467_2017_600_MOESM1_ESM.pdf]

**Description of Supplementary Files**

File Name: Supplementary Information
Description: Supplementary Figures, Supplementary Methods, Supplementary Tables, Supplementary Discussion and Supplementary References

File Name: Peer Review File

## SUPPLEMENTARY METHODS

**Proteomic analysis:** Purified nanoparticles and proteins were incubated at 95°C in 8M Urea for 5 minutes. The polymer was separated from the proteins by adding a 2-fold volume equivalent of acetonitrile and storing overnight at -20 °C. The proteins were pelleted at 10,000 *rcf* for 10 minutes and washed twice with cold acetonitrile.

*Reduction, Alkylation and Tryptic Digestion*

Protein pellets were resuspended in 8M urea and proteaseMAX surfactant (Promega) per manufacturer's instructions. Equivalent amounts of protein (20ug) from each condition were reduced (10 mM dithiothreitol, 56 °C for 45 min) and alkylated (50 mM iodoacetamide, room temperature in the dark for 1 h). Proteins were subsequently digested with trypsin (sequencing grade, Promega), at an enzyme/substrate ratio of 1:50, at room temperature overnight in 100 mM ammonium acetate pH 8.9. Trypsin activity was quenched by adding formic acid to a final concentration of 5%. Peptides were desalted using C18 SpinTips (Protea) then lyophilized and stored at −80 °C.

*Tandem Mass Tags (TMT) Labeling*

Peptides were labeled with TMT 10plex (Thermo) per manufacturer's instructions. Lyophilized samples were dissolved in 70 µL ethanol and 30 µl of 500 mM triethylammonium bicarbonate, pH 8.5, and the TMT reagent was dissolved in 30 µl of anhydrous acetonitrile. The solution containing peptides and TMT reagent was vortexed, incubated at room temperature for 1 h. Samples labeled with the ten different isotopic TMT reagents were combined and concentrated to completion in a vacuum centrifuge. Biological duplicates were analyzed in two sets of experiments, each with 3 technical triplicates. The samples were labeled using the TMT 10plex channels as presented in Supplementary Table 1:

***Supplementary Table 1*** **Samples and labels used in the TMT 10plex experiments**

| | **Biological replicate 1** | **Biological replicate 2** |
|---|---|---|
| **Isotopic label:** | PEG density of nanoparticle (ID) | PEG density of nanoparticle (ID) |
| **126** | **15** PEG chains per 100 $nm^2$ (50A) | **15** PEG chains per 100 $nm^2$ (50A) |
| **127N** | **25** PEG chains per 100 $nm^2$ (51A) | **25** PEG chains per 100 $nm^2$ (51A) |
| **127C** | **42** PEG chains per 100 $nm^2$ (52A) | **42** PEG chains per 100 $nm^2$ (52A) |
| **128N** | **15** PEG chains per 100 $nm^2$ (53A) | **15** PEG chains per 100 $nm^2$ (53A) |
| **128C** | **28** PEG chains per 100 $nm^2$ (54A) | **28** PEG chains per 100 $nm^2$ (54A) |
| **129N** | **18** PEG chains per 100 $nm^2$ (71A) | **18** PEG chains per 100 $nm^2$ (71A) |
| **129C** | **25** PEG chains per 100 $nm^2$ (72A) | **25** PEG chains per 100 $nm^2$ (72A) |
| **130N** | Reference plasma | Reference plasma |
| **130C** | Diluted plasma | Diluted plasma |
| **131** | Plasma | Plasma |

Each experiment included two independent batches of nanoparticles with 15 and 25 PEG chains per 100 $nm^2$. These nanoparticles therefore had a total of four biological replicates. All isotopic quantification data

was compared to the 130N channel. This reference contained plasma which was collected, and treated in the same manner as the nanoparticles: that is, through a 20-cm Sephacryl S-400 HR column (fractions 9-11 were collected) followed by three washing steps with PBS on a Vivaspin 1,000 kDa MWCO ultrafiltration unit. The other plasma channels (130C and 131) were plasma diluted 200-fold, and normal plasma, respectively. These samples were used to assess whether the size-exclusion column and ultrafiltration purification or dilution had any effect on the amounts of different proteins measured.

*LC-MS/MS*
Peptides (100ng) were loaded on a precolumn and separated by reverse phase HPLC (Thermo Easy nLC1000) over a 140 minute gradient before nanoelectrospray using a QExactive mass spectrometer (Thermo). The mass spectrometer was operated in a data-dependent mode. The parameters for the full scan MS were: resolution of 70,000 across 350-2000 *m/z*, AGC $3e^6$, and maximum IT 50 ms. The full MS scan was followed by MS/MS for the top 10 precursor ions in each cycle with a NCE of 32 and dynamic exclusion of 30 s. Each experiment was analyzed three times. Raw mass spectral data files (.raw) were searched using Proteome Discoverer 1.4 (Thermo) and Mascot version 2.4.1 (Matrix Science). Mascot search parameters were: 10 ppm mass tolerance for precursor ions; 0.8 Da for fragment ion mass tolerance; 2 missed cleavages of trypsin; fixed modification was carbamidomethylation of cysteine and TMT 10plex modification of lysines and peptide N-termini; variable modification was methionine oxidation. Only peptides with a Mascot score greater than or equal to 25 and an isolation interference less than or equal to 30 were included in the quantitative data analysis. A protein was considered identify if at least 2 unique peptides or 3 total peptides were identified. TMT quantification was obtained using Proteome Discoverer and isotopically corrected per manufacturer's instructions, and the values were normalized to the median of each channel.

*Statistical analysis*
All replicates (technical and biological) were used to evaluate the effect of PEG density on the adsorption of proteins. Linear regressions (on the curve obtained by plotting the relative increase in protein/peptide *vs*. the PEG density of the nanoparticles) were calculated by the GraphPad Prism 6.05 software using the means obtained from Proteome Discoverer, on all measurements. The slope of the curve was considered to be different from zero if the *p* value was below 0.05.

*ProteomeXchange:*
Submission details:
**Project Name:** TMT-Label Nanoparticles PEG density Bertrand et al. 2017
**Project accession:** PXD006043
**Project DOI:** Not applicable
Reviewer account details:
**Username:** reviewer75016@ebi.ac.uk
**Password:** Wc6nFEa2

**In vitro complement activation:** Blood from C57bl/6 mice was collected by cardiac punction and centrifuged at 2,000 rcf for 10 minutes. Serum was collected and incubated immediately with nanoparticles. In brief, 20 µL of nanoparticles (concentration 30 mg/mL) were added to 40 µL of serum, and incubated at 37°C for 1 hour. To stop the reaction, 60 µL of EDTA 25 mM was added, and samples were kept frozen until further analysis. The concentration of anaphylatoxin C5a in each sample was measured by using a mouse C5a ELISA (ab193718, Abcam, MA) per the manufacturer's protocol.

**In vivo complement activation:** Prior to the injection of nanoparticles, 50 uL of blood was collected by the saphenous vein, from healthy male Balb/c mice. Mice were injected intravenously with 3 mg of nanoparticles, and blood was collected in EDTA-coated centrifuge tubes, at 5, 15, 45 minutes. Plasma was separated by centrifugation (2,000 rcf for 10 minutes), and samples were frozen until further use. Complement activation was assayed by measuring the levels of terminal complement complex (C5b-9) using a mouse C5b-9 ELISA (EKU07600, Biomatik, Cambridge, ON) per the manufacturer's protocol.

## SUPPLEMENTARY DISCUSSION

### Self-assembly *vs*. surface grafting

In this work, the amount of PEG on the surface of nanoparticles is the result of the self-assembly of diblock copolymers, in the absence of surfactants or surface-stabilizing molecules. Since the hydrophilic polymer is present only in the polymer precursor solution, the amount of PEG in the system is defined by the length of each block of the copolymer (for example, 33 wt% PEG with $PEG_{5k}$-$PLGA_{10k}$ copolymers, 15.6 wt% with $PEG_{5k}$-$PLGA_{27k}$). We establish in Fig 1 that 1) most of the PEG is integrated into the nanoparticle, irrespective of the PLGA-PEG mixture used, and 2) most of it is solvated on the surface of the nanoparticle, irrespective of the nanoparticle diameter. Since the surface-to-volume ratio increases when nanoparticles get smaller, the PEG density on different particles prepared with the same PEG content decreases proportionally to their diameter. To study nanoparticles between 55 and 140 nm, mixtures of various uni- and diblock copolymer precursors were used to obtain a broad range of PEG contents, from 3.5 to 27 wt%.

In other studies, surface PEGylation is often carried out after synthesis of the core. For example, by conjugating reactive PEGs onto the surface[1, 2, 3, 4] or adsorbing surfactant molecules.[5, 6] In those cases, the final amount of PEG attached to the nanoparticle results from the ability of a PEG chain to interact with the surface. Given the hydrated and flexible nature of PEG chains, achieving high PEG densities requires minimizing steric hindrance and favoring interactions with surface of the core.[7] In that context, macromolecules can be more densely tethered to the surface of smaller nanoparticles.[2, 8] The assessment of the impact of size is therefore complicated, especially when indirect quantification methods are required to measure the PEG content (*e.g.*, measuring unreacted thiols,[3] or fluorescently labelling the polymer[4]). Together these differences in methodology and the different methods used for quantitation (radioactivity *vs*. anti-PEG ELISA and fluorescence) explain the discrepancies between our observations and those of others.

### Comparison with liposomes

While polymeric nanoparticles have a dense polymeric core, liposomes consist of phospholipid bilayers delimiting an aqueous center; most of their volume is occupied by a hydration solution, which is not taken into consideration when reporting lipid compositions. While depictions in wt% can be informative from a formulation perspective, they hardly offer any representation of surface density, which is potentially more amenable to comparisons between systems. To compare the polymeric nanoparticles studied here and liposomes, assumptions about the surface area occupied by each of the phospholipid molecule are needed. In the literature, this value depends notably on the chemical structure of the phospholipid and the presence of cholesterol in the bilayer.[9] Likewise, because of the presence of an aqueous compartment inside the liposomes, and depending on the liposome preparation method, assumptions about the amounts of PEG facing the internal compartment (and not actively granting steric protection[10] are also needed.

For example, a rough interval of PEG content can be obtained if we estimate the proportion of PEG facing outward to be 60-100%. Hence, 3-6 mol% (vs. total amount of lipid) of DSPE-PEG(5000) would be equivalent to a threshold of 20 PEG chains per 100 nm2 for hypothetical liposomes prepared with dimyristoylphosphatidylcholine (DMPC, 678 g/mol, specific area 44 Å2), 40 mol% cholesterol (387 g/mol), and DSPE-PEG(5000) (5750 g/mol). These values are in agreement with those found in the literature for long-circulating liposomes prepared with PEG5k.[11, 12, 13] Nevertheless, we would emphasize that comparisons with commercially available PEGylated doxorubicin liposomes might be precarious, since the length of the PEG used in this work differs from that found in those formulations (5,000 vs. 2,000, respectively).

**Choice of TMT-label quantitative proteomics analysis vs. label-free proteomics.**

In literature focusing on the study of interactions between nanoparticles and proteins[14, 15], researchers mainly use label-free quantification to determine the relative contribution each protein to the overall corona covering nanoparticles (Protein A represents X% of the total proteins found in sample 1). Due to differences in molecular weights, trypsin digestion patterns, ionization and the number of unique peptides, not all proteins are equal with respect to the signal provided by MS. Therefore, in label-free proteomics, quantification methods are used to determine the relative amounts of each protein compared to others, notably using spectral counting[16] and other algorithms.[17, 18]

In the present work, quantitative protein analysis was chosen instead using TMT-labels. In this method, samples are labeled with different isotopic tags before being injected together in the instrument.[19] This method makes comparisons between the amounts of individual proteins in each sample more accurate (*i.e.*, protein A is more abundant in sample 1 than in sample 2). However, the quantification algorithms discussed above have not been as extensively studied with TMT-labeling (and other quantitative proteomic techniques). In other words, no widely-accepted methodology currently exists to highlight quantitative differences between samples, together with the relative importance of one protein compared to another. It is beyond the scope of this study to define new methodologies to do so.

Our choice of using TMT-labels was guided by challenges in isolating PEGylated nanoparticles from free proteins in plasma using centrifugation methodologies commonly used in the field.[20] Using the radioactive label in the particles, it was determined that only a small proportion (< 25%) of the PLGA-PEG nanoparticles could be pelleted using high centrifugation speeds (> 28,000 rcf). We therefore relied on size exclusion columns[21] and ultrafiltration techniques to separate nanoparticles from the bulk of free proteins. Nevertheless, in our hands, these methods did not allow to get rid of all free proteins; it was observed that control plasma, treated with the same methodology, afforded a small amount of residual proteins (i.e., reference plasma). TMT-analysis, which compared the quantities of protein in samples containing nanoparticles relative to reference plasma, allowed to determine how PEG density affected the adsorption of proteins without interference from the residual proteins.

*Supplementary Table 2* **Polymer characterization data.**

| | Origin | nLA | mGA | $M_n$ PLGA Block | $M_w/M_n$ | PEG content (wt%) |
|---|---|---|---|---|---|---|
| **PLAG20k** | In Lab | 162 | 143 | 20,000[a] | 1.57 | 0 |
| **PLGA30k** | Lactel, Cupertino, CA | 249 | 230 | 29,000[a] | 1.88 | 0 |
| **PLGA95k** | Corbion Purac, Lenexa, KS | 817 | 754 | 95,000[a] | 1.97 | 0 |
| **PLGA5k-PEG5k** | In Lab | 36 | 35 | 4,622[b] | 1.13 | 52 |
| **PLGA10k-PEG5k** | In Lab | 52 | 57 | 7,122[b] | 1.18 | 41 |
| **PLGA27k-PEG5k** | Boehringer Ingelheim, Ridgefield, CT | 218 | 214 | 28,146[b] | 1.47 | 15 |

[a]Determined by GPC in tetrahydrofuran (relative to poly(styrene standards) [b]Determined by $^1$H-NMR

*Supplementary Table 3* **Nanoparticle preparation and characterization data**

| | Precursor solution (wt%) | | | | | | | | | | | | | | | |
|---|---|---|---|---|---|---|---|---|---|---|---|---|---|---|---|---|
| | Uniblock | | | Diblock | | | | | | | | | | | | |
| Reference | PLAG20k | PLGA30k | PLGA95k | PLGA5k-PEG5k | PLGA10k-PEG5k | PLGA27k-PEG5k | Volume of water (mL) | Stirring speed (rpm) | Mean (Z average n=3) | | SD Z-average (n=3) | Mean PDI (n=3) | | SD PDI | PEG content (wt%) | PEG chains per 100 $nm^2$ |
| 55-nm | | | | | | | | | | | | | | | | |
| rNP-3 | | 10 | | | | 90 | MF device | | 59 | ± | 0.57 | 0.062 | ± | 0.018 | 9.7% | 14 |
| rNP-7 | | | | | | 100 | MF device | | 53 | ± | 0.15 | 0.078 | ± | 0.006 | 12.6% | 16 |
| 026 | 5 | | | | | 95 | 10 | 800 | 56 | ± | 0.64 | 0.130 | ± | 0.005 | 13.1% | 18 |
| ZNP-001 | | | | 20 | | 80 | 7.5 | 1600 | 56 | ± | 0.40 | 0.084 | ± | 0.009 | 16.8% | 22 |
| 008 | | | | | 17 | 83 | 10 | 1600 | 58 | ± | 0.27 | 0.151 | ± | 0.005 | 18.5% | 26 |
| 005 | | 80 | | | 20 | | 10 | 1600 | 57 | ± | 0.44 | 0.175 | ± | 0.011 | 19.5% | 27 |
| 022 | | | | | 50 | 50 | 10 | 1400 | 56 | ± | 0.15 | 0.151 | ± | 0.007 | 25.4% | 34 |
| 023 | | | | 45 | | 55 | 10 | 1600 | 54 | ± | 0.46 | 0.170 | ± | 0.01 | 27.5% | 35 |
| 067 | | | | | 50 | 55 | 10 | 1400 | 57 | ± | 0.77 | 0.168 | ± | 0.002 | 25.9% | 36 |
| 068 | | | | | 55 | 45 | 10 | 1400 | 57 | ± | 0.57 | 0.144 | ± | 0.011 | 27.1% | 37 |
| 90- nm | | | | | | | | | | | | | | | | |
| 021 | 55 | 15 | | | | 30 | 10 | 1400 | 94 | ± | 0.43 | 0.155 | ± | 0.015 | 4.2% | 9 |
| 024 | 55 | 15 | | | | 30 | 10 | 1400 | 94 | ± | 1.16 | 0.110 | ± | 0.01 | 5.2% | 12 |
| 047 | 35 | 20 | | | | 45 | 10 | 1400 | 89 | ± | 0.57 | 0.129 | | 0.028 | 6.3% | 13 |
| 074 | 22.5 | 27.5 | | | | 50 | 10 | 1200 | 91 | ± | 0.96 | 0.175 | ± | 0.01 | 6.7% | 15 |
| 053 | 22.5 | 27.5 | | | | 50 | 10 | 1000 | 91 | ± | 0.32 | 0.120 | ± | 0.014 | 6.8% | 15 |
| 061 | 22.5 | 27.5 | | | | 50 | 10 | 1000 | 91 | ± | 1.47 | 0.109 | ± | 0.008 | 6.9% | 15 |
| 066 | 22.5 | 27.5 | | | | 55 | 10 | 1200 | 89 | ± | 1.19 | 0.117 | ± | 0.012 | 7.1% | 15 |
| 063 | 22.5 | 27.5 | | | | 50 | 10 | 1000 | 90 | ± | 1.52 | 0.147 | ± | 0.001 | 7.1% | 15 |
| 071 | | 40 | | | | 60 | 10 | 1400 | 85 | ± | 1.15 | 0.106 | ± | 0.007 | 8.4% | 17 |
| rNP-2 | | 30 | | | | 70 | MF device | | 93 | ± | 2.15 | 0.093 | ± | 0.015 | 7.8% | 17 |
| 055 | | 40 | | | | 60 | 10 | 1400 | 89 | ± | 0.81 | 0.111 | ± | 0.018 | 8.2% | 18 |
| 057 | | 40 | | | | 60 | 10 | 1400 | 85 | ± | 0.33 | 0.103 | ± | 0.014 | 8.6% | 18 |
| 044 | | 40 | | | | 60 | 10 | 1400 | 92 | ± | 0.27 | 0.165 | ± | 0.011 | 8.4% | 19 |
| 045 | | 40 | | | | 60 | 10 | 1400 | 90 | ± | 0.48 | 0.141 | | 0.01 | 8.7% | 19 |
| 046 | | 10 | 10 | | | 80 | 10 | 1200 | 83 | ± | 1.60 | 0.127 | | 0.009 | 10.0% | 21 |
| 059 | | 10 | 10 | | | 80 | 10 | 1200 | 89 | ± | 0.90 | 0.138 | ± | 0.018 | 10.2% | 22 |
| 041 | | 20 | | | | 80 | 10 | 800 | 85 | ± | 0.37 | 0.102 | ± | 0.011 | 11.1% | 23 |
| 042 | | 20 | | | | 80 | 10 | 1000 | 89 | ± | 0.37 | 0.116 | ± | 0.008 | 10.8% | 23 |
| 043 | | 10 | | | | 90 | 10 | 600 | 82 | ± | 0.51 | 0.156 | ± | 0.01 | 11.9% | 24 |
| 072 | | | 20 | 5 | | 75 | 10 | 1400 | 84 | ± | 0.60 | 0.107 | ± | 0.008 | 11.9% | 24 |
| 056 | | | 20 | 5 | | 75 | 10 | 1400 | 93 | ± | 0.88 | 0.163 | ± | 0.02 | 11.0% | 25 |
| 051 | | | 20 | | 5 | 75 | 9 | 1400 | 86 | ± | 3.57 | 0.140 | | 0.02 | 11.6% | 25 |
| rNP-8 | | | 10 | | | 90 | MF device | | 93 | ± | 1.06 | 0.117 | ± | 0.012 | 11.2% | 25 |
| 065 | | | 20 | 5 | | 75 | 10 | 1400 | 86 | ± | 0.95 | 0.106 | ± | 0.011 | 12.4% | 26 |
| 060 | | | 20 | 5 | | 75 | 10 | 1400 | 86 | ± | 0.67 | 0.123 | ± | 0.006 | 12.5% | 26 |
| 031 | | | 20 | 7.3 | | 72.7 | 10 | 1200 | 89 | ± | 0.38 | 0.149 | ± | 0.016 | 13.1% | 28 |
| 028 | | | 20 | 7.3 | | 72.7 | 10 | 1000 | 90 | ± | 0.42 | 0.167 | ± | 0.005 | 13.1% | 28 |
| 016 | | 45 | | | 55 | | 10 | 1600 | 85 | ± | 0.36 | 0.143 | ± | 0.005 | 20.0% | 41 |
| 073 | 45 | | | | | 55 | 10 | 1600 | 88 | ± | 0.16 | 0.179 | ± | 0.011 | 19.7% | 42 |
| 052 | | 45 | | | 55 | | 9 | 1800 | 96 | ± | 0.27 | 0.092 | ± | 0.02 | 19.3% | 45 |
| 048 | | 45 | | 55 | | | 10 | 1800 | 95 | ± | 0.36 | 0.070 | | 0.012 | 19.7% | 45 |
| 062 | | 45 | | | 55 | | 10 | 1800 | 103 | ± | 1.45 | 0.132 | ± | 0.006 | 20.4% | 50 |
| 058 | | 45 | | | 55 | | 9 | 1800 | 100 | ± | 0.57 | 0.104 | ± | 0.01 | 20.9% | 50 |

*Supplementary Table 3 (continued)*

| Reference | PLAG20k | PLGA30k | PLGA95k | PLGA5k-PEG5k | PLGA10k-PEG5k | PLGA27k-PEG5k | Volume of water (mL) | Stirring speed (rpm) | | Mean (Z average n=3) | | SD Z-average (n=3) | Mean PDI (n=3) | | SD PDI | PEG content (wt%) | PEG chains per 100 $nm^2$ |
|---|---|---|---|---|---|---|---|---|---|---|---|---|---|---|---|---|---|
| 140-nm | | | | | | | | | | | | | | | | | |
| 020 | | 92 | | | 8 | | 10 | 1400 | | 136 | ± | 1.40 | 0.102 | ± | 0.02 | 3.5% | 11 |
| 034 | | 19 | 50 | | | 31 | 10 | 1600 | | 145 | ± | 0.64 | 0.138 | ± | 0.005 | 3.4% | 12 |
| 032 | | 30 | 45 | | | 25 | 10 | 1600 | | 143 | ± | 0.78 | 0.126 | ± | 0.009 | 3.7% | 13 |
| 033 | | 22 | 50 | | | 28 | 10 | 1600 | | 139 | ± | 0.69 | 0.141 | ± | 0.019 | 3.8% | 13 |
| 040 | | 10 | 44 | | | 36 | 10 | 1400 | | 134 | ± | 0.50 | 0.111 | ± | 0.028 | 5.6% | 18 |
| 038 | | | 60 | | | 40 | 10 | 1200 | | 136 | ± | 1.36 | 0.127 | ± | 0.011 | 5.8% | 19 |
| 035 | | | 56 | | | 44 | 10 | 1600 | | 136 | ± | 1.25 | 0.171 | ± | 0.026 | 6.3% | 21 |
| 069 | | | 55 | | | 55 | 10 | 1000 | | 129 | ± | 2.30 | 0.101 | ± | 0.019 | 7.0% | 22 |
| 018 | | | 60 | | | 40 | 10 | 800 | | 144 | ± | 1.72 | 0.106 | ± | 0.021 | 6.3% | 22 |
| 037 | | | 50 | | | 50 | 10 | 1000 | | 138 | ± | 1.00 | 0.131 | ± | 0.007 | 7.0% | 23 |
| 030 | | 60 | 15 | | 25 | | 10 | 1200 | | 134 | ± | 0.95 | 0.137 | ± | 0.012 | 9.7% | 31 |
| 027 | | 60 | 15 | | 25 | | 10 | 800 | | 133 | ± | 1.33 | 0.090 | ± | 0.021 | 9.9% | 32 |
| 019 | | 35 | 35 | | 30 | | 10 | 1400 | | 138 | ± | 1.28 | 0.187 | ± | 0.003 | 10.6% | 35 |
| 070 | | 35 | 35 | | 30 | | 10 | 1400 | | 152 | ± | 3.76 | 0.093 | ± | 0.041 | 11.1% | 40 |

***Supplementary Table 4*** **Number of animals in each group.** Sample sizes were chosen to obtain sufficient biological repeats while ensuring feasibility of the study; key groups were repeated to confirm data when needed ($n > 5$). Healthy animals were used without randomization, and investigators were not blinded. Animals were excluded if experimental problems occurred (problem during injection or sampling, animal death upon dosing ($n = 1$)).

**Figure 2** — **Balb/c mice**

| | PEG density | # animals |
|---|---|---|
| 55-nm nanoparticles | 14 | 3 |
| | 16 | 3 |
| | 18 | 4 |
| | 22 | 4 |
| | 26 | 4 |
| | 27 | 4 |
| | 34 | 4 |
| | 35 | 4 |
| 90-nm nanoparticles | 12 | 4 |
| | 15 | 4 |
| | 17 | 4 |
| | 19 | 5 |
| | 22 | 3 |
| | 23 | 4 |
| | 25 | 3 |
| | 28 | 5 |
| | 41 | 5 |
| 140-nm nanoparticles | 11 | 3 |
| | 13 | 5 |
| | 18 | 5 |
| | 21 | 3 |
| | 22 | 4 |
| | 23 | 5 |
| | 32 | 5 |
| | 35 | 3 |

**Figure 3** — **C57bl/6**

| | PEG density | # animals |
|---|---|---|
| 90-nm NP | 15 | 11 |
| | 18 | 8 |
| | 25 | 8 |
| | 45 | 7 |

C3$^{-/-}$ — B6;129S4-C3$^{tm1Crr}$/J

| | PEG density | # animals |
|---|---|---|
| 90-nm NP | 15 | 10 |
| | 18 | 5 |
| | 25 | 4 |
| | 45 | 5 |

ApoE$^{-/-}$ — B6;129P2-Apoe$^{tm1Unc}$/J

| | PEG density | # animals |
|---|---|---|
| 90-nm NP | 15 | 5 |
| | 18 | 4 |
| | 25 | 4 |
| | 45 | 5 |

C57Bl/6 — with clusterin

| | PEG density | # animals |
|---|---|---|
| 90-nm NP | 15 | 5 |
| | 18 | 5 |
| | 25 | 4 |
| | 45 | 5 |

LDLR$^{-/-}$ — B6;129S7-LDLR$^{tm1Her}$/J

| | PEG density | # animals |
|---|---|---|
| 90-nm NP | 15 | 5 |
| | 18 | 5 |
| | 25 | 5 |
| | 45 | 5 |

**Supplementary Figure 3**

*Same as Figure 1*

**Supplementary Figure 4**

C57bl/6

| | PEG density | # animals |
|---|---|---|
| 90-nm NP | 13 | 5 |
| | 15 | 11 |
| | 18 | 8 |
| | 19 | 5 |
| | 22 | 5 |
| | 25 | 8 |
| | 28 | 5 |
| | 45 | 7 |

**Supplementary Figure 5**

Sprague-Dawley rats

| | PEG density | # animals |
|---|---|---|
| 55-nm | 36 | 3 |
| | 37 | 3 |
| 90-nm | 15 | 3 |
| | 17 | 3 |
| | 26 | 3 |
| | 50 | 3 |
| 140-nm | 22 | 3 |
| | 40 | 3 |

**Supplementary Figure 6 and 7**

| | | |
|---|---|---|
| 90-nm NP | 15 | 2 |
| | 18 | 2 |
| | 25 | 2 |
| | 45 | 2 |

**Supplementary Figure 8**

C57Bl/6 + C57Bl/6 plasma

| | PEG density | # animals |
|---|---|---|
| 90-nm | 15 | 5 |
| | 45 | 5 |

C57bl/6 + ApoE$^{-/-}$ plasma

| | PEG density | # animals |
|---|---|---|
| 90-nm NP | 15 | 4 |

C57bl/6 + LDLR$^{-/-}$ ApoB$^{only}$ plasma

| | PEG density | # animals |
|---|---|---|
| 90-nm | 15 | 5 |
| | 45 | 4 |

**Supplementary Figure 9**

C57Bl/6 — with PCSK9

| | PEG density | # animals |
|---|---|---|
| 90-nm NP | 15 | 4 |
| | 18 | 3 |
| | 25 | 4 |
| | 45 | 5 |

***Supplementary Table 5*** **Raw data for circulation profiles obtained in C57Bl/6 and KO animals.**

| | C57bl/6 | | | | C3$^{-/-}$ | | | | ApoE$^{-/-}$ | | | |
|---|---|---|---|---|---|---|---|---|---|---|---|---|
| | PEG density (PEG chains per 100 nm$^2$) | | | | PEG density (PEG chains per 100 nm$^2$) | | | | PEG density (PEG chains per 100 nm$^2$) | | | |
| **Time (min)** | **15 (n=11)** | **18 (n=10)** | **25 (n=8)** | **45 (n=7)** | **15 (n=5)** | **18 (n=5)** | **25 (n=4)** | **45 (n=5)** | **15 (n=5)** | **18 (n=4)** | **25 (n=4)** | **45 (n=5)** |
| 15 | **52.5** ± 5.9 | **62.3** ± 7.9 | **70.0** ± 7.8 | **68.5** ± 13.5 | **64.7** ± 12.0 | **68.2** ± 4.3 | **72.5** ± 10.0 | **78.2** ± 7.5 | **57.5** ± 9.8 | **59.9** ± 14.8 | **77.0** ± 7.9 | **66.3** ± 7.1 |
| 30 | **36.5** ± 8.5 | **55.2** ± 6.7 | **64.7** ± 6.9 | **58.9** ± 6.3 | **51.0** ± 10.0 | **64.0** ± 14.3 | **66.6** ± 10.6 | **71.8** ± 9.1 | **40.9** ± 16.0 | **54.2** ± 13.2 | **73.2** ± 8.8 | **62.2** ± 6.5 |
| 60 | **24.2** ± 8.9 | **45.5** ± 6.1 | **56.9** ± 6.5 | **56.6** ± 12.5 | **33.8** ± 5.3 | **50.2** ± 9.0 | **61.0** ± 10.7 | **64.5** ± 8.0 | **13.0** ± 6.1 | **37.4** ± 14.0 | **65.5** ± 10.6 | **54.9** ± 6.5 |
| 120 | **14.3** ± 6.9 | **31.4** ± 6.0 | **50.4** ± 5.1 | **48.7** ± 8.0 | **23.1** ± 3.1 | **36.9** ± 7.1 | **53.7** ± 7.6 | **56.1** ± 8.1 | **4.6** ± 1.2 | **22.5** ± 9.1 | **53.7** ± 6.4 | **51.1** ± 4.2 |
| 240 | **6.1** ± 2.3 | **15.1** ± 2.5 | **39.0** ± 5.2 | **40.4** ± 5.0 | **11.6** ± 0.9 | **20.1** ± 9.8 | **44.1** ± 4.3 | **46.2** ± 6.5 | **2.5** ± 0.5 | **8.0** ± 2.6 | **37.9** ± 5.4 | **42.7** ± 4.4 |
| 360 | **2.5** ± 1.2 | **5.4** ± 1.2 | **23.4** ± 6.0 | **29.3** ± 2.7 | **5.0** ± 1.0 | **7.1** ± 0.9 | **24.4** ± 5.2 | **34.3** ± 3.8 | **1.3** ± 0.3 | **3.0** ± 0.9 | **21.3** ± 3.5 | **31.4** ± 3.9 |
| AUC$_{0-6h}$ (%ID.h.g$^{-1}$) | 87 ± 25.7 | 161 ± 21.5 | 257 ± 32.4 | 273 ± 39.9 | 132 ± 16.8 | 190 ± 43 | 291 ± 39.3 | 309 ± 29.1 | 59 ± 13.6 | 124 ± 40 | 288 ± 30.7 | 286 ± 27.5 |
| Ke (h$^{-1}$) | 0.75 ± 0.21 | 0.41 ± 0.03 | 0.18 ± 0.04 | 0.13 ± 0.02 | 0.42 ± 0.05 | 0.37 ± 0.02 | 0.17 ± 0.01 | 0.12 ± 0.02 | 1.19 ± 0.07 | 0.51 ± 0.02 | 0.22 ± 0.01 | 0.12 ± 0.02 |

| | Clusterin pre-incubation | | | | LDLR$^{-/-}$ | | | | PCSK9 treatment | | | |
|---|---|---|---|---|---|---|---|---|---|---|---|---|
| | PEG density (PEG chains per 100 nm$^2$) | | | | PEG density (PEG chains per 100 nm$^2$) | | | | PEG density (PEG chains per 100 nm$^2$) | | | |
| **Time (min)** | **15 (n=5)** | **18 (n=5)** | **25 (n=4)** | **45 (n=4)** | **15 (n=5)** | **18 (n=5)** | **25 (n=5)** | **45 (n=4)** | **15 (n=4)** | **18 (n=3)** | **25 (n=4)** | **45 (n=5)** |
| 15 | **69.3** ± 6.1 | **70.7** ± 2.8 | **60.3** ± 11.0 | **61.1** ± 3.0 | **73.9** ± 6.2 | **86.2** ± 8.8 | **84.6** ± 8.3 | **78.4** ± 4.2 | **63.9** ± 3.7 | **71.2** ± 5.6 | **79.5** ± 9.6 | **75.9** ± 5.1 |
| 30 | **58.1** ± 4.5 | **62.4** ± 6.5 | **57.8** ± 10.5 | **55.5** ± 1.7 | **63.2** ± 6.0 | **79.0** ± 4.7 | **80.3** ± 9.9 | **72.0** ± 3.0 | **55.2** ± 3.4 | **65.5** ± 5.0 | **73.5** ± 6.1 | **68.9** ± 3.9 |
| 60 | **44.7** ± 4.3 | **54.5** ± 8.9 | **53.7** ± 9.5 | **51.2** ± 1.4 | **43.2** ± 5.6 | **67.6** ± 5.8 | **71.0** ± 6.6 | **62.5** ± 3.1 | **44.8** ± 2.9 | **55.7** ± 3.2 | **65.8** ± 5.9 | **65.7** ± 3.2 |
| 120 | **27.2** ± 3.7 | **41.5** ± 3.0 | **48.3** ± 8.4 | **46.8** ± 1.6 | **26.6** ± 3.1 | **48.5** ± 3.8 | **61.4** ± 4.7 | **56.4** ± 3.9 | **28.8** ± 1.5 | **40.9** ± 3.6 | **57.8** ± 4.1 | **58.1** ± 4.1 |
| 240 | **10.5** ± 1.7 | **17.8** ± 2.3 | **38.9** ± 7.7 | **40.9** ± 0.7 | **11.7** ± 1.5 | **23.9** ± 2.2 | **51.1** ± 4.8 | **53.5** ± 4.1 | **10.9** ± 1.3 | **18.1** ± 0.6 | **47.6** ± 4.4 | **48.8** ± 4.2 |
| 360 | **3.6** ± 0.8 | **6.4** ± 1.5 | **28.8** ± 4.8 | **33.2** ± 1.8 | **4.9** ± 0.6 | **9.0** ± 0.8 | **30.2** ± 3.7 | **37.7** ± 2.1 | **4.1** ± 0.4 | **7.4** ± 0.8 | **32.9** ± 2.5 | **42.4** ± 3.8 |
| AUC$_{0-6h}$ (%ID.h.g$^{-1}$) | 147 ± 15.7 | 195 ± 17 | 264 ± 47.8 | 267 ± 5.7 | 152 ± 16.0 | 242 ± 18.7 | 340 ± 30.9 | 333 ± 17.6 | 147 ± 6.0 | 198 ± 12 | 322 ± 27.1 | 331 ± 22.8 |
| Ke (h$^{-1}$) | 0.50 ± 0.03 | 0.41 ± 0.04 | 0.14 ± 0.00 | 0.11 ± 0.01 | 0.46 ± 0.02 | 0.37 ± 0.01 | 0.17 ± 0.01 | 0.11 ± 0.00 | 0.52 ± 0.01 | 0.39 ± 0.02 | 0.14 ± 0.00 | 0.09 ± 0.01 |

| | C57Bl/6 pre-incubation | | | | ApoE$^{-/-}$ pre-incubation | | | | LDLR$^{-/-}$ApoB100$^{only}$ pre-incubation | | | |
|---|---|---|---|---|---|---|---|---|---|---|---|---|
| | PEG density (PEG chains per 100 nm$^2$) | | | | PEG density (PEG chains per 100 nm$^2$) | | | | PEG density (PEG chains per 100 nm$^2$) | | | |
| **Time (min)** | **15 (n=5)** | **18 (n=0)** | **25 (n=0)** | **45 (n=5)** | **15 (n=4)** | **18 (n=0)** | **25 (n=0)** | **45 (n=0)** | **15 (n=5)** | **18 (n=0)** | **25 (n=0)** | **45 (n=4)** |
| 15 | **57.7** ± 9.9 | | | **70.0** ± 10.0 | **41.2** ± 6.8 | | | | **50.5** ± 5.7 | | | **67.5** ± 5.3 |
| 30 | **47.3** ± 5.1 | | | **57.0** ± 15.8 | **34.1** ± 6.3 | | | | **50.7** ± 13.7 | | | **61.7** ± 5.7 |
| 60 | **34.6** ± 2.4 | **Not done** | | **57.1** ± 8.5 | **24.9** ± 3.7 | **Not done** | | | **34.5** ± 8.4 | **Not done** | | **49.9** ± 13.4 |
| 120 | **21.7** ± 1.2 | | | **50.0** ± 6.9 | **15.8** ± 1.9 | | | | **22.1** ± 3.7 | | | **51.9** ± 4.2 |
| 240 | **7.3** ± 0.5 | | | **47.3** ± 5.9 | **6.9** ± 1.7 | | | | **7.8** ± 1.7 | | | **45.3** ± 3.7 |
| 360 | **2.4** ± 0.1 | | | **33.8** ± 7.1 | **2.7** ± 0.3 | | | | **2.3** ± 0.6 | | | **35.5** ± 2.5 |
| AUC$_{0-6h}$ (%ID.h.g$^{-1}$) | 115 ± 6.8 | | | 294 ± 38.4 | 87.1 ± 7.5 | | | | 115 ± 20.4 | | | 290 ± 26.2 |
| Ke (h$^{-1}$) | 0.54 ± 0.02 | | | 0.12 ± 0.03 | 0.54 ± 0.05 | | | | 0.54 ± 0.02 | | | 0.1 ± 0.00 |

**Supplementary figures.**

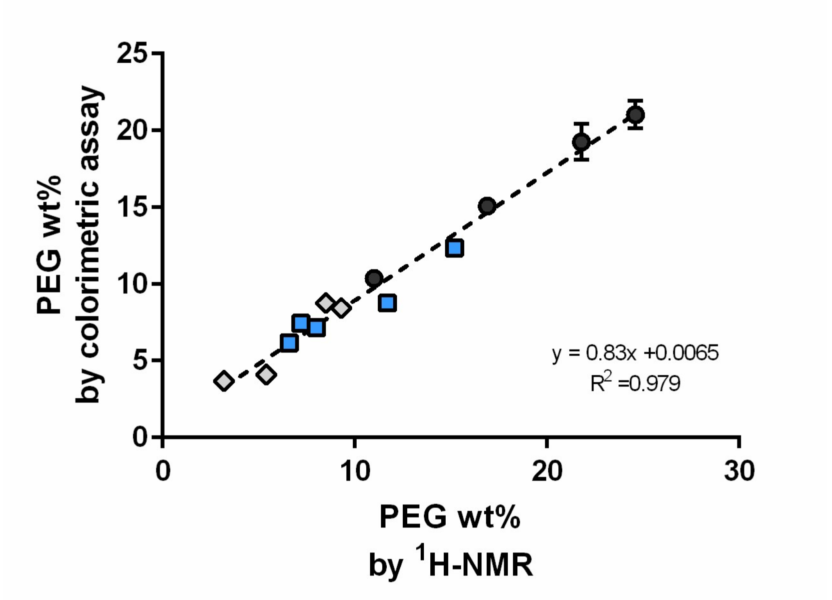


**Supplementary Figure 1 PEG content results obtained by 1H-NMR are in good agreement with those obtained by a broadly used iodine-based colorimetric assay**. PEGylated nanoparticles were hydrolyzed with 1N sodium hydroxide for 30 minutes and reacted with an iodine/potassium iodine aqueous solution (9mM iodine and 155 mM of potassium iodine). Absorbance at 495 nm was read on a Tecan Infinite 500 plate reader. The PEG content was determined by comparing absorbance of samples to a PEG calibration curve.[22]

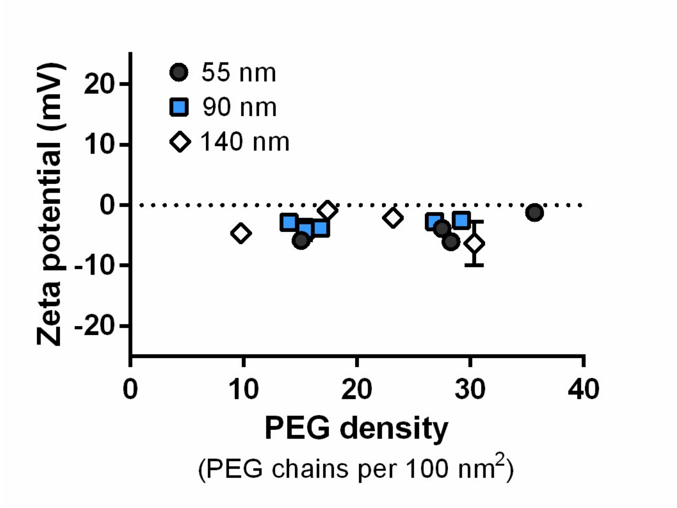


**Supplementary Figure 2** Nanoparticles prepared with mixtures of PEG-PLGA and PLGA all show similar zeta-potential, irrespective of size or PEG density.

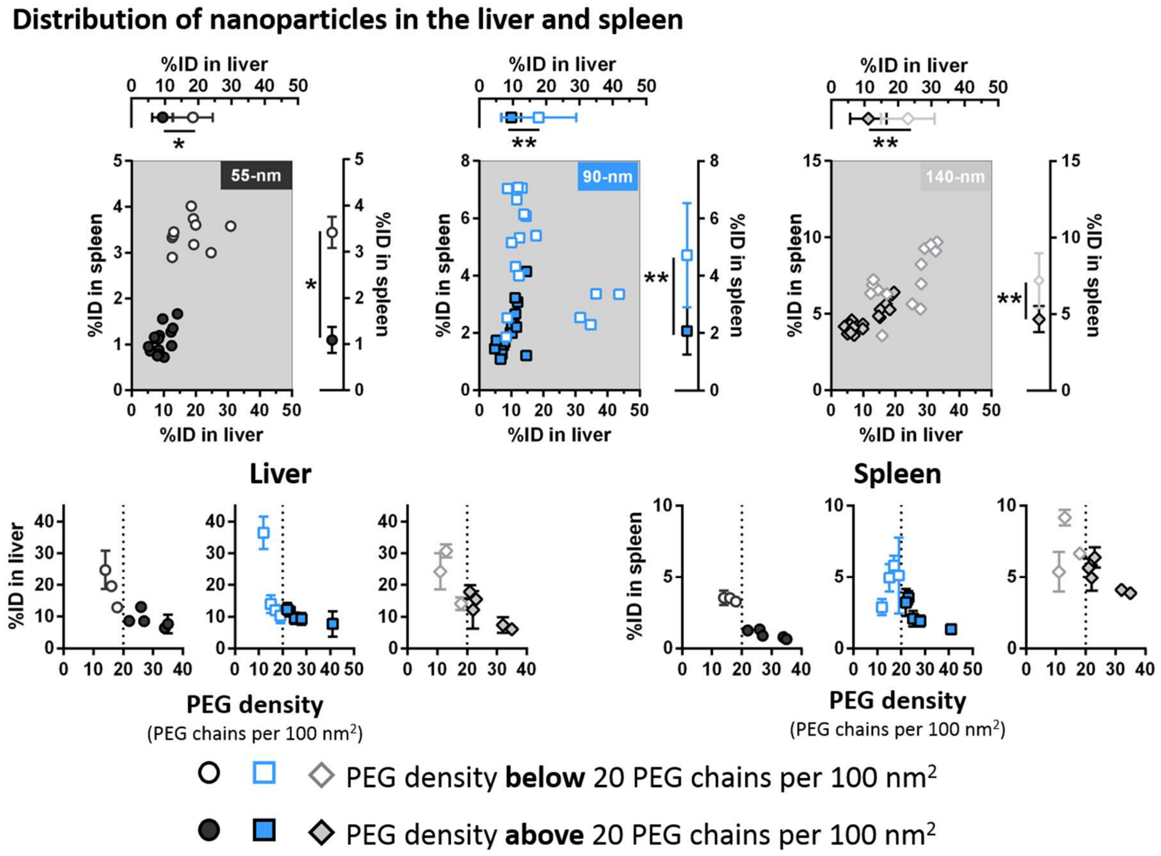


**Supplementary Figure 3 Nanoparticles with PEG densities below the 20 PEG chains per 100 nm² show higher distribution to the liver and spleen.** While liver distribution of highly PEGylated nanoparticles is similar across sizes, capture in the spleen seems to increase with the nanoparticle diameter. In each of the main graphs, each point represents one animal, while means ± SD are presented offset. * $p < 0.05$ as determined by t-test, ** $p < 0.05$ as determined by Mann-Whitney U (non-parametric) test.

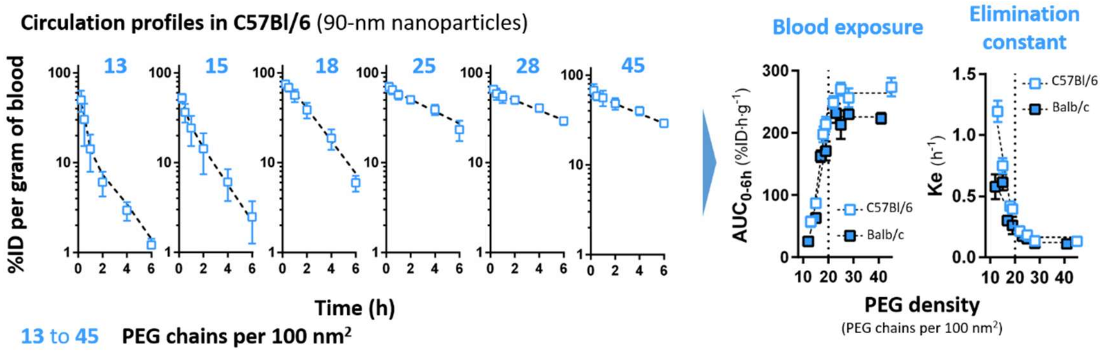


**Supplementary Figure 4 A PEG density threshold is also perceivable in C57Bl/6 mice.** As seen in Balb/c mice, the circulation profile and pharmacokinetic parameters of 90-nm nanoparticles in C57Bl/6 mice show little added benefit when the PEG density is augmented above 20 PEG chains per 100 nm². Values are means ± SD ($n$= 4-11). For comparison purposes, full symbols represent the pharmacokinetic parameters for Balb/c mice.

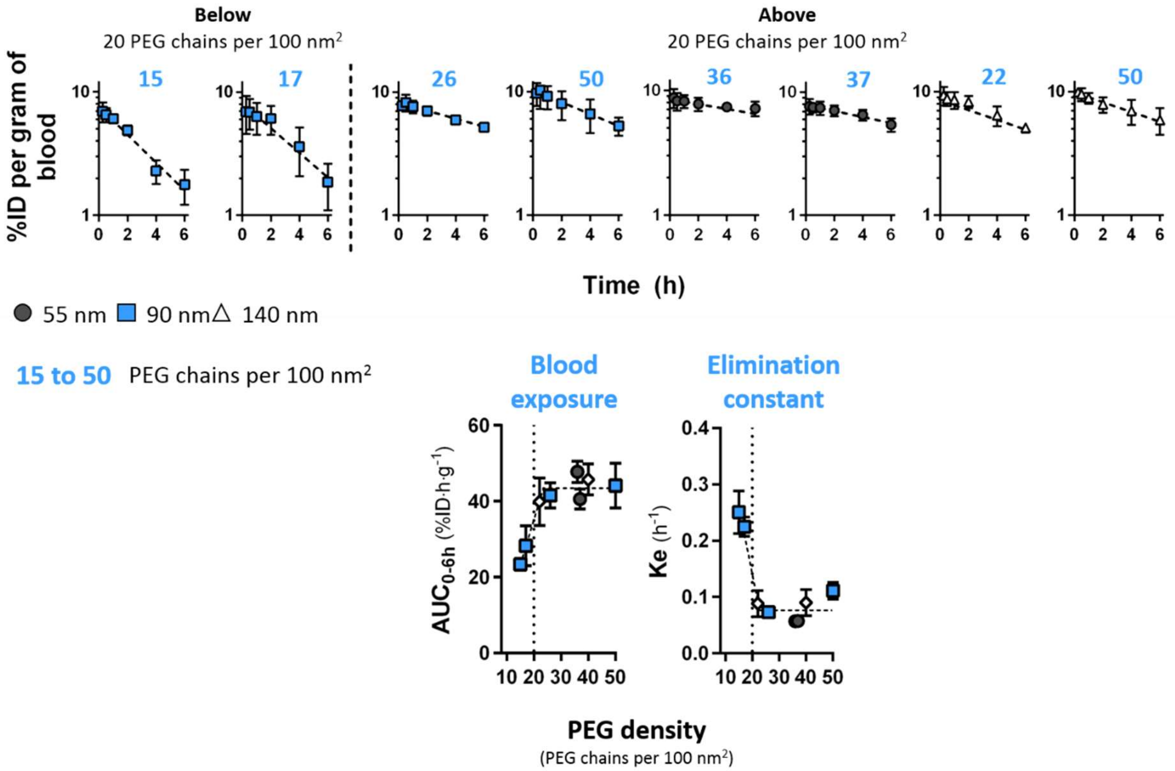


**Supplementary Figure 5 A PEG density threshold is also perceivable in Sprague-Dawley rats; above 20 PEG chains per 100 nm$^2$ the circulation profiles of nanoparticles with different diameters are comparable.** Values are means ± SD (*n*= 3).

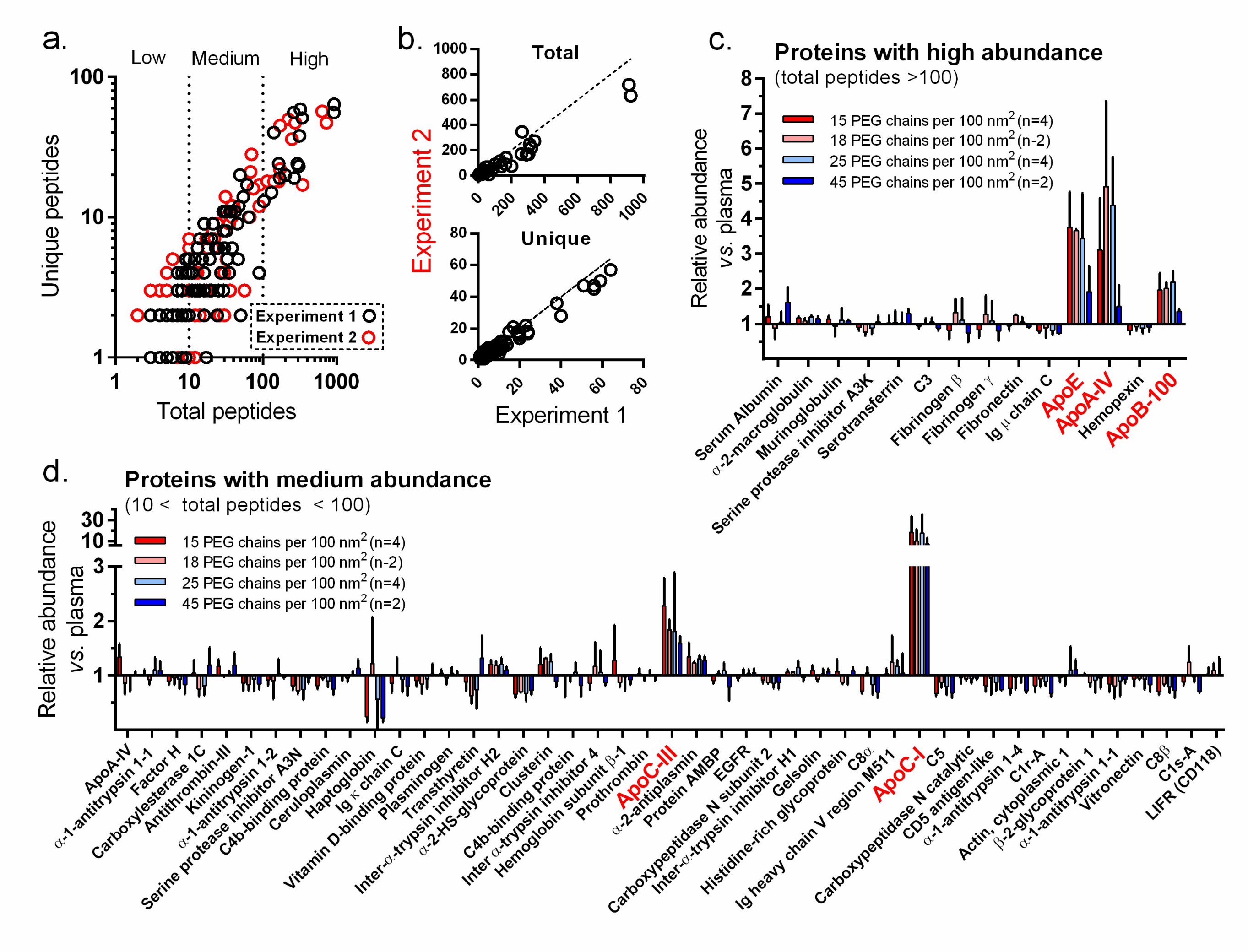


**Supplementary Figure 6 Quantitative proteomics analysis identified 5 proteins that are enriched on the surface of all nanoparticles. A.** The abundance of all proteins (high, medium, low) was categorized depending on the total number of peptides identified by LC-MS. For each protein (each symbol), the total number of peptides was correlated with the number of unique peptides. **B**. The numbers of total and unique peptides identified for each protein were similar between both biological replicates. **C**. Among the most abundant proteins found in the samples, ApoE, ApoA-IV and ApoB-100 are more than 2-fold more abundant on the surface of the nanoparticles than in plasma. **D**. Among the proteins with medium abundance, ApoC-I and ApoC-III are enriched on the surface of all nanoparticles. Values are means ± SD (*n*= 2-4 biological replicates, 3 technical replicates).

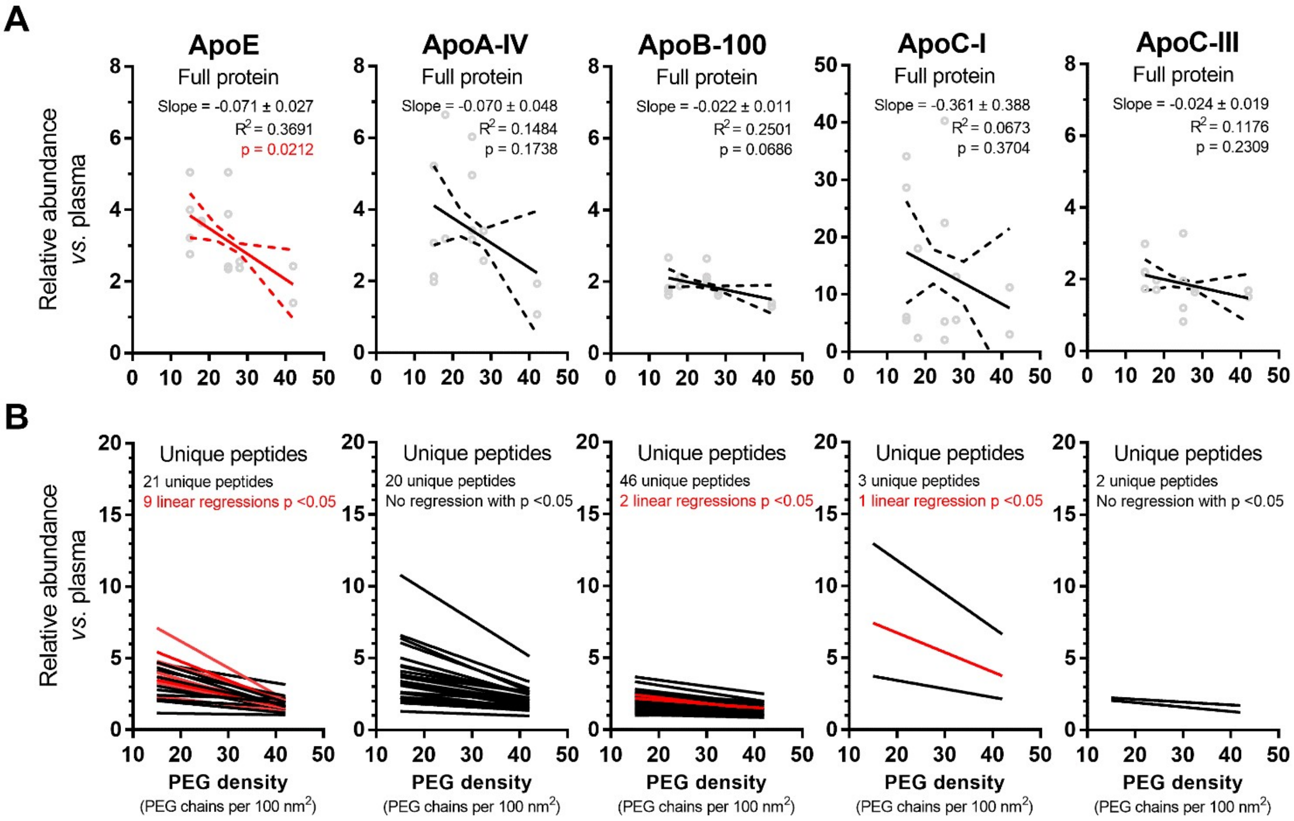


**Supplementary Figure 7 ApoE is the only protein which quantities appear to be inversely correlated to the PEG density.** Linear regressions on the amounts of protein (**A**) and unique peptides (**B**) highlight how PEG density of the nanoparticles influences the adsorption of proteins. ApoE is the only protein for which PEG surface density affects adsorption. Dots represent individual measurements, red lines show linear regressions which are statistically different from zero, dotted lines show the 95% confidence interval for the regression.

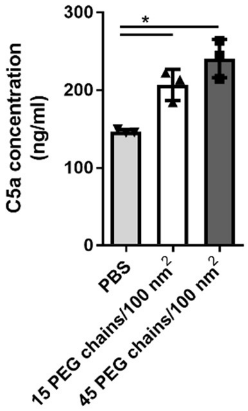


**Supplementary Figure 8** Nanoparticles with low and high PEG densities (fast and rapid blood clearance, respectively) are mild activators of the complement cascade, irrespective of the PEG density ($n$= 3, * $p$ < 0.05 as determined by one-way ANOVA and Tukey's *post-hoc*).

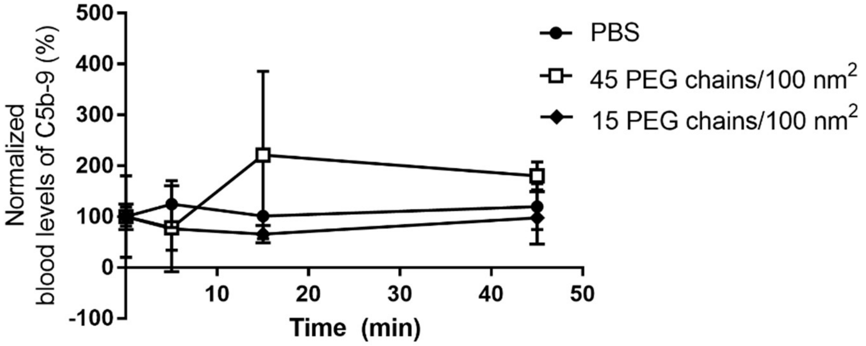


**Supplementary Figure 9** Intravenous injections of nanoparticles with low and high PEG densities (fast and rapid blood clearance, respectively) fail to significantly increase the circulating levels of the terminal complex of the complement cascade (C5b-9) in Babl/c mice ($n$= 3).

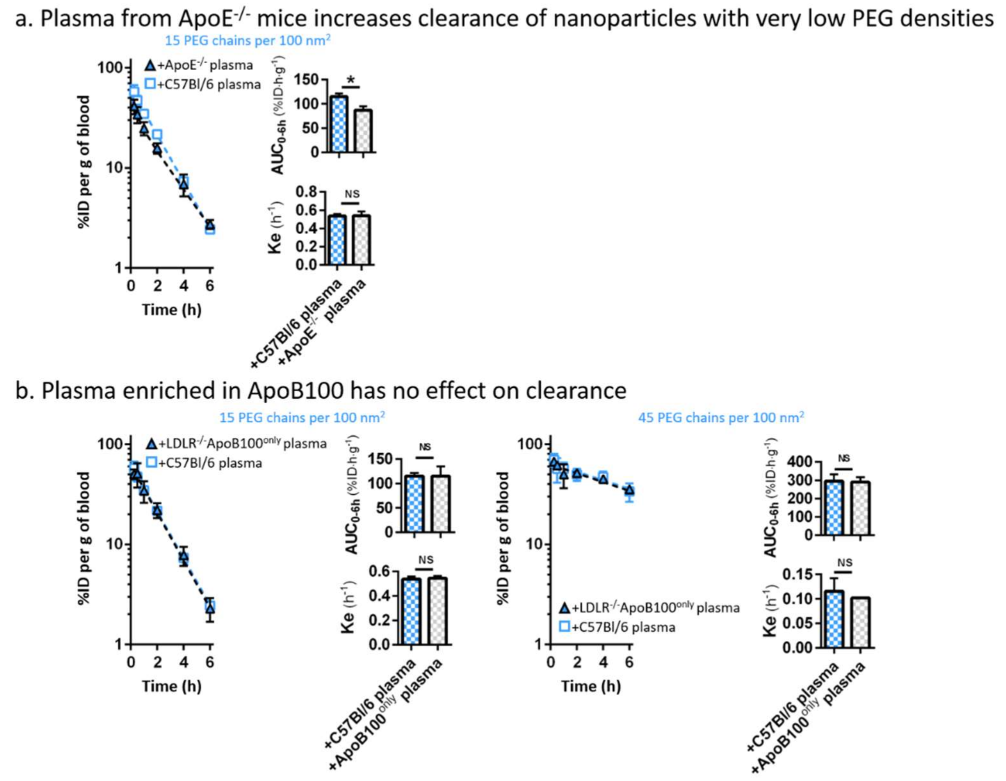


**Supplementary Figure 10 The role of apolipoproteins is evidenced by pre-incubation of nanoparticles with plasma from transgenic animals. A.** On nanoparticles with very low PEG densities, the lack of ApoE increases the clearance, highlighting the role of the protein as a possible dysopsonin. **B.** Incubation with plasma containing 10-fold the levels of ApoB100 has no effect on the clearance of nanoparticles with low and high PEG densities. Values represent means ± SD ($n$= 4-5). * $p < 0.05$ as determined by t-test.

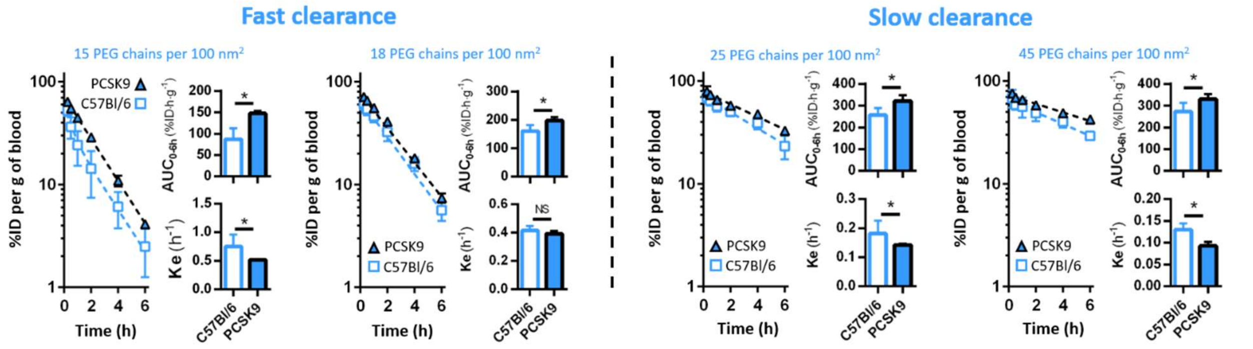


**Supplementary Figure 11 Animals administered with PCSK9 60 minutes before dosing with nanoparticles show reduced clearance.** These observations further highlight the involvement of LDLR in the clearance of nanoparticles. It also suggests that the effect seen in LDLR$^{-/-}$ animals is not mediated by indirect mechanisms resulting from an increase in the lipid burden. Values represent means ± SD ($n$= 4-5). * $p < 0.05$ as determined by t-test.

## SUPPLEMENTARY REFERENCES


1. Perrault SD, Walkey C, Jennings T, Fischer HC, Chan WC. Mediating tumor targeting efficiency of nanoparticles through design. *Nano Lett* **9**, 1909-1915 (2009).

2. Yang Q, Jones SW, Parker CL, Zamboni WC, Bear JE, Lai SK. Evading Immune Cell Uptake and Clearance Requires PEG Grafting at Densities Substantially Exceeding the Minimum for Brush Conformation. *Mol Pharmaceutics* **11**, 1250-1258 (2014).

3. Walkey CD, Olsen JB, Guo H, Emili A, Chan WCW. Nanoparticle Size and Surface Chemistry Determine Serum Protein Adsorption and Macrophage Uptake. *J Am Chem Soc* **134**, 2139-2147 (2012).

4. Perry JL*, et al.* PEGylated PRINT Nanoparticles: The Impact of PEG Density on Protein Binding, Macrophage Association, Biodistribution, and Pharmacokinetics. *Nano Lett* **12**, 5304-5310 (2012).

5. Hamad I, Al-Hanbali O, Hunter AC, Rutt KJ, Andresen TL, Moghimi SM. Distinct polymer architecture mediates switching of complement activation pathways at the nanosphere-serum interface: implications for stealth nanoparticle engineering. *ACS Nano* **11**, 6629-6638 (2010).

6. Kulkarni SA, Feng S-S. Effects of Particle Size and Surface Modification on Cellular Uptake and Biodistribution of Polymeric Nanoparticles for Drug Delivery. *Pharm Res* **30**, 2512-2522 (2013).

7. Kingshott P, Thissen H, Griesser HJ. Effects of cloud-point grafting, chain length, and density of PEG layers on competitive adsorption of ocular proteins. *Biomaterials* **23**, 2043-2056 (2002).

8. Hill HD, Millstone JE, Banholzer MJ, Mirkin CA. The Role Radius of Curvature Plays in Thiolated Oligonucleotide Loading on Gold Nanoparticles. *ACS Nano* **3**, 418-424 (2009).

9. Hung W-C, Lee M-T, Chen F-Y, Huang HW. The Condensing Effect of Cholesterol in Lipid Bilayers. *Biophys J* **92**, 3960-3967 (2007).

10. Nakamura K, Yamashita K, Itoh Y, Yoshino K, Nozawa S, Kasukawa H. Comparative studies of polyethylene glycol-modified liposomes prepared using different PEG-modification methods. *BBA -Biomembranes* **1818**, 2801-2807 (2012).

11. Mori A, Klibanov AM, Torchilin VP, Huang L. Influence of the steric barrier activity of amphiphatic poly(ethylene glycol) and ganglioside GM1 on the circulation time of the liposomes and on the target binding of immunoliposomes *in vivo*. *FEBS Lett* **284**, 263-266 (1991).

12. Klibanov AL, Maruyama K, Beckerleg AM, Torchilin VP, Huang L. Activity of amphipathic poly(ethylene glycol) 5000 to prolong the circulation time of liposomes depends on the liposome size and is unfavorable for immunoliposome binding to target. *BBA -Biomembranes* **1062**, 142-148 (1991).

13. Klibanov AL, Maruyama K, Torchilin VP, Huang L. Amphipathic polyethyleneglycols effectively prolong the circulation time of liposomes. *FEBS Lett* **268**, 235-237 (1990).

14. Tenzer S*, et al.* Rapid formation of plasma protein corona critically affects nanoparticle pathophysiology. *Nat Nanotechnol* **8**, 772-781 (2013).

15. Schöttler S*, et al.* Protein adsorption is required for stealth effect of poly(ethylene glycol)- and poly(phosphoester)-coated nanocarriers. *Nat Nanotechnol* **11**, 372-377 (2016).

16. Monopoli MP*, et al.* Physical−Chemical Aspects of Protein Corona: Relevance to in Vitro and in Vivo Biological Impacts of Nanoparticles. *J Am Chem Soc* **133**, 2525-2534 (2011).

17. Silva JC, Gorenstein MV, Li G-Z, Vissers JPC, Geromanos SJ. Absolute Quantification of Proteins by LCMSE : A Virtue of Parallel ms Acquisition. *Molecular & Cellular Proteomics* **5**, 144-156 (2006).

18. Ishihama Y*, et al.* Exponentially Modified Protein Abundance Index (emPAI) for Estimation of Absolute Protein Amount in Proteomics by the Number of Sequenced Peptides per Protein. *Molecular & Cellular Proteomics* **4**, 1265-1272 (2005).

19. Dayon L*, et al.* Relative Quantification of Proteins in Human Cerebrospinal Fluids by MS/MS Using 6-Plex Isobaric Tags. *Anal Chem* **80**, 2921-2931 (2008).

20. Docter D*, et al.* Quantitative profiling of the protein coronas that form around nanoparticles. *Nat Protocols* **9**, 2030-2044 (2014).

21. Cedervall T*, et al.* Understanding the nanoparticle-protein corona using methods to quantify exchange rates and affinities of proteins for nanoparticles. *Proc Natl Acad Sci USA* **104**, 2050-2055 (2007).

22. Bazile D, Prud'homme C, Bassoullet MT, Marlard M, Spenlehauer G, Veillard M. Stealth Me. PEG-PLA Nanoparticles Avoid Uptake by the Mononuclear Phagocytes System. *J Pharm Sci* **84**, 493-498 (1995).
